# Leiomyosarcoma Arising in the Pancreatic Duct: A Case Report and Review of the Current Literature

**DOI:** 10.1155/2010/252364

**Published:** 2010-06-09

**Authors:** Nicole D. Riddle, Brian C. Quigley, Irwin Browarsky, Marilyn M. Bui

**Affiliations:** ^1^Department of Pathology and Cell Biology, University of South Florida College of Medicine, 12901 Bruce B. Downs Blvd, MDC 11, Tampa, FL 33612, USA; ^2^Departments of Anatomic Pathology and Sarcoma, H. Lee Moffitt Cancer Center & Research Institute, Tampa, FL 33612, USA

## Abstract

*Context*. Leiomyosarcomas are rare malignant smooth muscle tumors that may arise in any organ or tissue that contains smooth muscle, commonly within the gastrointestinal tract. They are most often found in the stomach, large and small intestines, and retroperitoneum. Primary pancreatic leiomyosarcoma is extremely rare, and to the best of our knowledge only 30 cases have been reported in the world literature since 1951. Our case represents the first to have a clear origin from the main pancreatic duct. *Case Report*. This case was diagnosed in a large, tertiary care center in Tampa, Florida. Pertinent information was obtained from chart review and interdepartmental collaboration. A mass in the tail of the pancreas was identified with large pleomorphic and spindle-shaped cells. Immunohistochemistry for vimentin, smooth muscle actin, and desmin was positive. All remaining immunohistochemical markers performed were negative. The tumor clearly originated from the pancreatic duct wall, filled and expanded the duct lumen, and was covered with a layer of benign biliary epithelium. *Conclusion*. Leiomyosarcoma of the pancreas is an extremely rare malignancy with few reported cases in the literature. The prognosis is poor, and treatment consists of alleviating symptoms and pain management. To our knowledge, this represents the first reported case demonstrating clear origin of a leiomyosarcoma from the pancreatic duct.

## 1. Introduction

Leiomyosarcomas are rare malignant tumors of smooth muscle origin that may arise in any organ or tissue that contains smooth muscle and comprise less than 1% of all cancers and 2%–9% of sarcomas [1]. They are most commonly found in the stomach and small intestine and may also be commonly found in the large intestine, uterus, and retroperitoneum [1]. Primary pancreatic leiomyosarcoma is extremely rare and has seldomly been reported in the literature. Typically the prognosis is poor, and treatment consists of alleviating symptoms and managing discomfort with one or more modalities including surgery, radiation, and/or chemotherapy. This case shows a clear origination from the pancreatic duct.

## 2. Case Report

**Figure 1:**
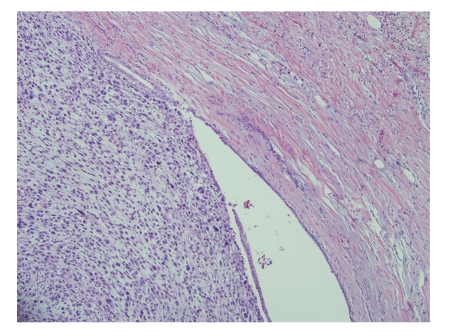
Low power view of the tumor showing the expansion of the duct wall with dilation of the lumen (H & E 100x).

**Figure 2:**
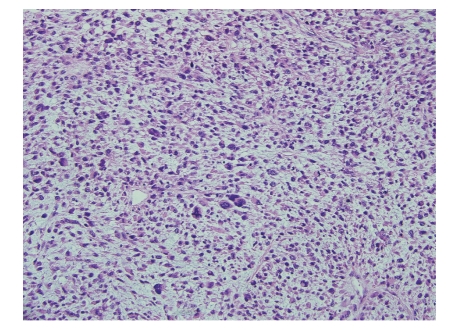
High power view showing highly pleomorphic cells and numerous/atypical mitosis (H & E 400x).

**Figure 3:**
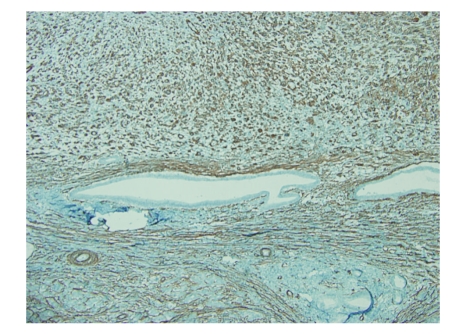
Smooth muscle actin immunohistochemical stain shows positive staining in the pleomorphic and spindle tumor cells, indicating smooth muscle origin of this lesion (100x).

**Figure 4:**
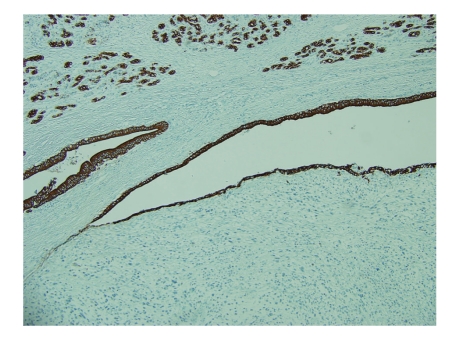
Pan-keratin, highlighting the residual ductal epithelium with no staining in tumor cells (100x).

An 83-year-old woman presented with a 3-month history of left-sided abdominal pain and weight loss. Her history was pertinent for a total abdominal hysterectomy with bilateral salpingo-oophorectomy in 1963 for benign leiomyomas with dysmenorrhea, a cholecystectomy in 1978 for cholelithiasis, and a hernia repair in 1993. No mass lesion was identified on physical examination. Abdominal CT scan and MRI revealed an 8 cm mass within the tail of the pancreas with no evidence of invasion into adjacent structures. At this time the presumed diagnosis was pancreatic adenocarcinoma. Ultrasound-guided biopsy of the lesion was attempted but yielded no diagnostic tissue. A distal pancreatectomy with splenectomy was performed and revealed an intrapancreatic tan, nodular mass measuring 8.5 × 7.0 × 6.2 cm with focal hemorrhagic areas suggestive of necrosis. The margins were grossly free of tumor. Histological examination showed large pleomorphic and spindle-shaped cells, focal necrosis, and a mitotic count of > 20 per 10 high power fields, with negative surgical margins (Figures 1 and 2). Multiple sections failed to demonstrate a malignant epithelial component. Immunohistochemical analysis showed tumor cells positive for smooth muscle actin (SMA), desmin, and vimentin and negative for other markers performed (pan cytokeratin, c-kit, CD34, and S-100) (Figures 3 and 4). There was a clear origination from the pancreatic duct wall with expansion of the lumen. Based on the histopathology and immunohistochemical profile, a diagnosis of leiomyosarcoma was rendered. The patient decided against further treatment and at 8 months was doing well with no apparent residual disease or metastasis.

## 3. Discussion

Primary pancreatic sarcomas of any type are rare although many have been reported in the literature including fibromyxoid sarcoma, follicular dendritic cell sarcoma, Kaposi's sarcoma, leiomyosarcoma, fibrosarcoma, liposarcoma, angiosarcoma, and rhabdomyosarcoma [2–14]. Epidemiologic data derived from case reports suggests that most of the cases of primary pancreatic sarcoma occur in patients over 50 if not over 70 years of age, except for rhabdomyosarcoma which favors children and sometimes young adults [8]. It has been suggested that the pancreatic duct or blood vessel walls most likely serve as an origin for pancreatic leiomyosarcomas, a thought that is supported by their usual location within the body or tail [15]. Our case represents the first time clear origination from the pancreatic duct wall has been shown.

Leiomyosarcomas are malignant smooth muscle neoplasms that can arise in any anatomic location containing smooth muscle, most commonly the stomach, but can be found anywhere along the gastrointestinal tract, as well as the uterus and retroperitoneum. They have also been reported in the skin, bladder, ovaries, salivary glands, larynx, gallbladder, adrenal glands, broad ligament, diaphragm, breast, vulva, penis, scrotum and testis, and, of course, the pancreas [16–37]. Leiomyosarcomas are rare, comprising less than 1% of all cancers. A compilation of data on pancreatic leiomyosarcomas presented by Aihara et al. shows an age range of 14–80 years for initial diagnosis of pancreatic leiomyosarcoma with a mean of 52.8 years and a median of 52.5 years [15]. Of the patients who died from pancreatic leiomyosarcoma, mean time to death was 11.5 months with a range of 5 days to 4 years [15]. These aggressive tumors tend to metastasize hematogenously, most commonly to the lung and less frequently the liver, brain, bone, spinal column, and skin [21, 31, 38]. On histopathological examination, malignancy is mainly determined by tumor necrosis, the number of mitoses (generally believed to be ≥ 2/10 HPF), atypical mitotic activity, and an infiltrative pattern. Most leiomyosarcomas are immunopositive for SMA, desmin, caldesmon, and vimentin. 

## 4. Conclusion

Leiomyosarcoma of the pancreas is an extremely rare malignancy with few reported cases in the literature. The prognosis is poor, and treatment consists of surgical resection with chemotherapy and/or radiation for alleviating symptoms and pain management. To our knowledge, this represents the first reported case demonstrating clear origin of a leiomyosarcoma from the pancreatic duct.
